# Synthesis of Humin-Phenol-Formaldehyde Adhesive

**DOI:** 10.3390/polym9080373

**Published:** 2017-08-18

**Authors:** Shimin Kang, Jinxia Fu, Gang Zhang, Wentao Zhang, Huibin Yin, Yongjun Xu

**Affiliations:** 1 Guangdong Provincial Key Laboratory of Distributed Energy Systems, Dongguan University of Technology, Dongguan 523808, China; kangshimin@dgut.edu.cn (S.K.); 2015804@dgut.edu.cn (G.Z.); yinhb@dgut.edu.cn (H.Y.); 2 Hawaii Natural Energy Institute, University of Hawaii, Honolulu, HI 96822, USA; jinxiafu@hawaii.edu; 3 Dongguan Cleaner Production Center, Dongguan University of Technology, Dongguan 523808, China; zhangwt@dgut.edu.cn

**Keywords:** humins, hydrothermal treatment, phenol-formaldehyde adhesive, hydrolysis

## Abstract

Humins are low-value-added byproducts from the biomass acid hydrolysis process. In the present work, humins were first employed as a phenol replacement for synthesis of modified phenol-formaldehyde adhesives through a two-step process. In this process, humins were first utilized to obtain alkaline soluble products, mainly consisting of phenolics, through a hydrothermal process. The obtained alkaline soluble products then reacted with phenol and formaldehyde to produce humin-phenol-formaldehyde adhesive (HPFA). The physicochemical properties of HPFA, including viscosity, bonding strength, pH, free formaldehyde level, free phenol level and solid content, met the requirements of the GB/T 14732-2006 Chinese National Standard.

## 1. Introduction

With the depletion of fossil energy source and the growing concerns over climate change, renewable energy and green chemicals derived from biomass have attracted much attention in the past few decades. The acid-catalyzed hydrolysis process has been demonstrated as a potential pathway to produce top platform chemicals, such as levulinic acid and 5-hydroxymethylfurfural, from biomass [1,2,3]. This process, however, inevitably generated byproducts mainly consisting of humins [4,5]. Humins, a class of carbon-rich solid residues [6,7], can fatally decrease the efficiency of biomass carbon utilization. It has been reported that 30–50 wt % of cellulose carbon finally formed humins in the hydrothermal treatment process for levulinic acid production [8]. It is, therefore, essential to develop value-added applications of humins for biorefineries.

Phenol-formaldehyde (PF) adhesive is one of the most widely used wood adhesives. The conventional pathways for PF adhesive production, however, still rely on petroleum-based phenol, which has been recognized as a major obstacle for green PF adhesive production. Recently, utilization of alternative and cheaper phenolic sources for PF resin production has been of great interest to biorefineries [9]. Renewable sources, such as lignin and biomass pyrolysis oils, were employed to replace petroleum phenol for PF adhesive production [10,11,12]. As lignin, humins are low-value-added polymers, which can be degraded and dissolve in alkaline solution [7,13]. The soluble characteristic of humins in alkaline conditions illustrates that they can be utilized as an alternative source for value-added chemical production.

In this article, a two-step reaction pathway was developed to synthetize humin-phenol-formaldehyde adhesive (HPFA) using humins. Humins were initially degraded to soluble products, and the obtained phenolic products further reacted with formaldehyde by partially substituting the phenol groups. The physicochemical properties of the HPFA were measured and compared with the GB/T 14732-2006 Chinese National Standard [14].

## 2. Methods

### 2.1. Alkaline Hydrothermal Treatment

The humins were obtained from a routine glucose acid hydrolysis [7]. The alkaline catalytic hydrothermal reaction was conducted in a 80 mL Teflon^®^ reactor (Zhengzhou Hezhong Instrument Co., Ltd. Zhengzhou, China. 4.0 ± 0.1 g humins and 20 ± 0.1 mL 3 M NaOH solution were added in the reactor. The reactor was heated to 200 ± 2 °C in an air-circulated oven for approximately 60 min and then kept at 200 ± 2 °C for 12 h. After the reaction, the reactor was cooled down with water to obtain an alkaline-dissolved humins (ADH) solution.

### 2.2. Synthesis of Adhesive

The synthesis of adhesive was conducted by adding 4.0 ± 0.1 g humins derived ADH solution, 4.0 ± 0.1 g phenol, and 10 ± 0.1 mL 37 wt % formaldehyde in a 250 mL flask. The phenol-formaldehyde polycondensation reaction was conducted at 85 ± 2 °C for 75 ± 2 min, with a mechanical agitation speed of 200 ± 5 rpm.

### 2.3. Characterization and Analysis

Cured ADH and HPFA were obtained by drying the ADH and HPFA solution, respectively, at 100 °C to constant weight and used for a series of characterization and analysis. Thermogravimetric (TG) and derivative thermogravimetric (DTG) analyses were conducted under 20 mL/min N_2_ flow by a Netzsch 209F3 (NETZSCH-Gerätebau GmbH, Selb, Germany). A sample of about ~5 mg (humins, cured ADH, or cured HPFA) was placed in a Al_2_O_3_ sample pan and heated from 30 to 800 °C with a heating rate of 15 °C·min^−1^. The curing of ADH and HPFA was conducted by drying at 100 °C to constant weight.

Differential scanning calorimetry (DSC) analysis was conducted on a Netzsch 200F3 analyzer (NETZSCH-Gerätebau GmbH, Selb, Germany) under a 50 mL/min N_2_ stream. A sample (~5 mg HPFA) was placed in a Al_2_O_3_ sample pan and heated from 30 to 100 °C with a heating rate of 5 °C·min^−1^, and then from 100 °C with a heating rate of 2 °C·min^−1^.

The functional groups were analyzed by Fourier transform infrared spectroscopy (FT-IR) on a Tensor 27 (Bruker, Karlsruhe, Germany). The FT-IR samples were prepared according to the work done by Smith et al. [15], in which KBr pellets were used as an interference-free matrix for performing the FT-IR measurements. All FTIR transmission spectra were recorded in the wavenumber range of 4000 to 500 cm^−1^ and were composed of 64 scans with a resolution of 4 cm^−1^. The solid C^13^ nuclear magnetic resonance (NMR) analysis was conducted on an Avance III HD 500 MHz (Bruker, Karlsruhe, Germany). The samples (humins, cured ADH and cured HPFA) were directly tested without any pretreatment.

The ADH solution was extracted by methylene chloride and analyzed by gas chromatography mass spectrometer (GC/MS) with an Agilent J&W DB-624 column by Shimadzu QP 2010 Plus system (Kyoto, Japan). The temperature of the injector was set at 250 °C. The furnace temperature program was 50 °C (hold 2 min) and ramped to 260 °C (10 °C/min, hold 5 min). The compounds were identified by means of the NIST08 and NIST08s mass spectral data library.

The surface morphology of samples (humins, cured ADH and cured HPFA) was studied using a JEOL JSM-6701F environmental scanning electron microscopy (SEM) system (Tokyo, Japan). Before the SEM test, the samples were pretreated by coating with plasma gold plated.

The physicochemical properties of the adhesive, such as solid content, dynamic viscosity, free formaldehyde level, free phenol level, pH value, and adhesive strength, were determined according to the GB/T 14074-2006 Chinese National Standard.

## 3. Results and Discussion

### 3.1. Hydrothermal Treatment

In general, the major obstacle to modifying humins’ structure is that they cannot dissolve in regular aqueous solution [13,16]. Hydrothermal treatment, however, can help the humins completely dissolve in alkaline aqueous solutions and form a black homogeneous solution (pH = 13.2). When the pH value of the black solution was adjusted to 2, approximately 75 wt % of the alkaline dissolved humins (ADH) precipitated, and these even precipitates cannot dissolve in the weak acid or neutral solutions anymore. In addition, the precipitated ADH exhibits two important characteristics: (1) most of these precipitates are water-insoluble, large-molecular-weight compounds, and (2) alkaline condition is preferred for further homogeneous modification of humins or ADH.

**Figure 1:**
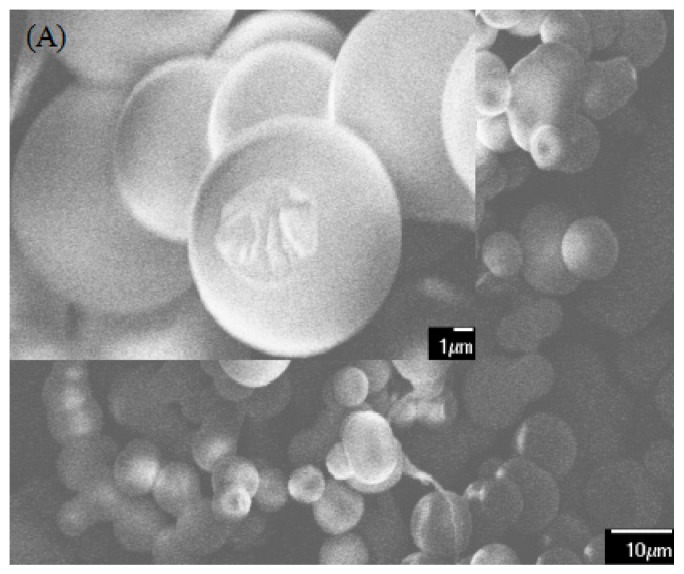

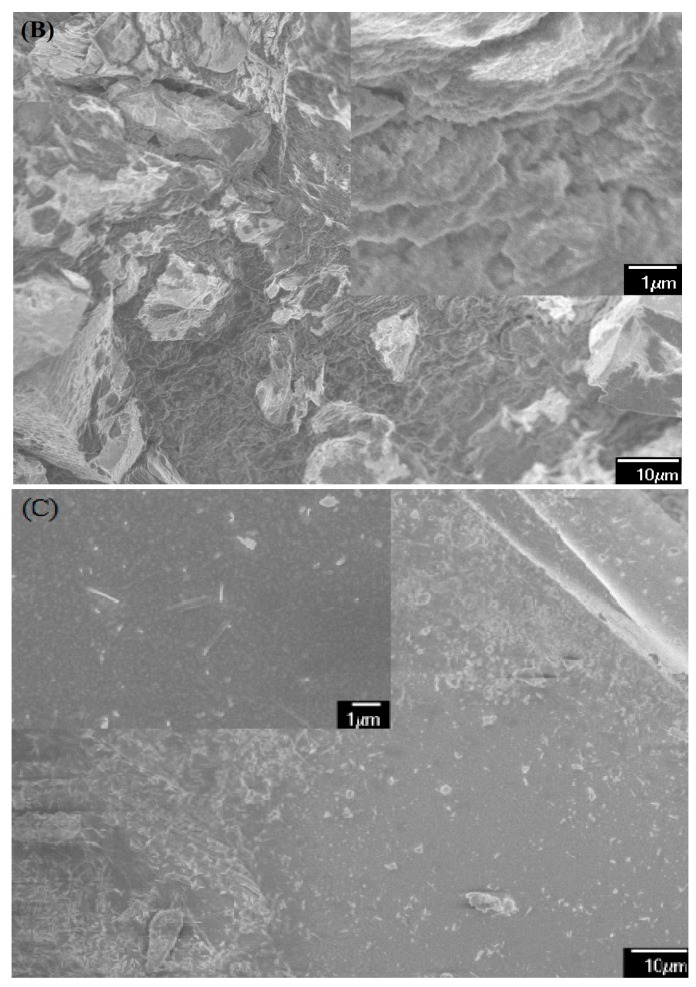
SEM images of humins (**A**), cured ADH (**B**), and cured HPFA (**C**).

**Figure 2:**
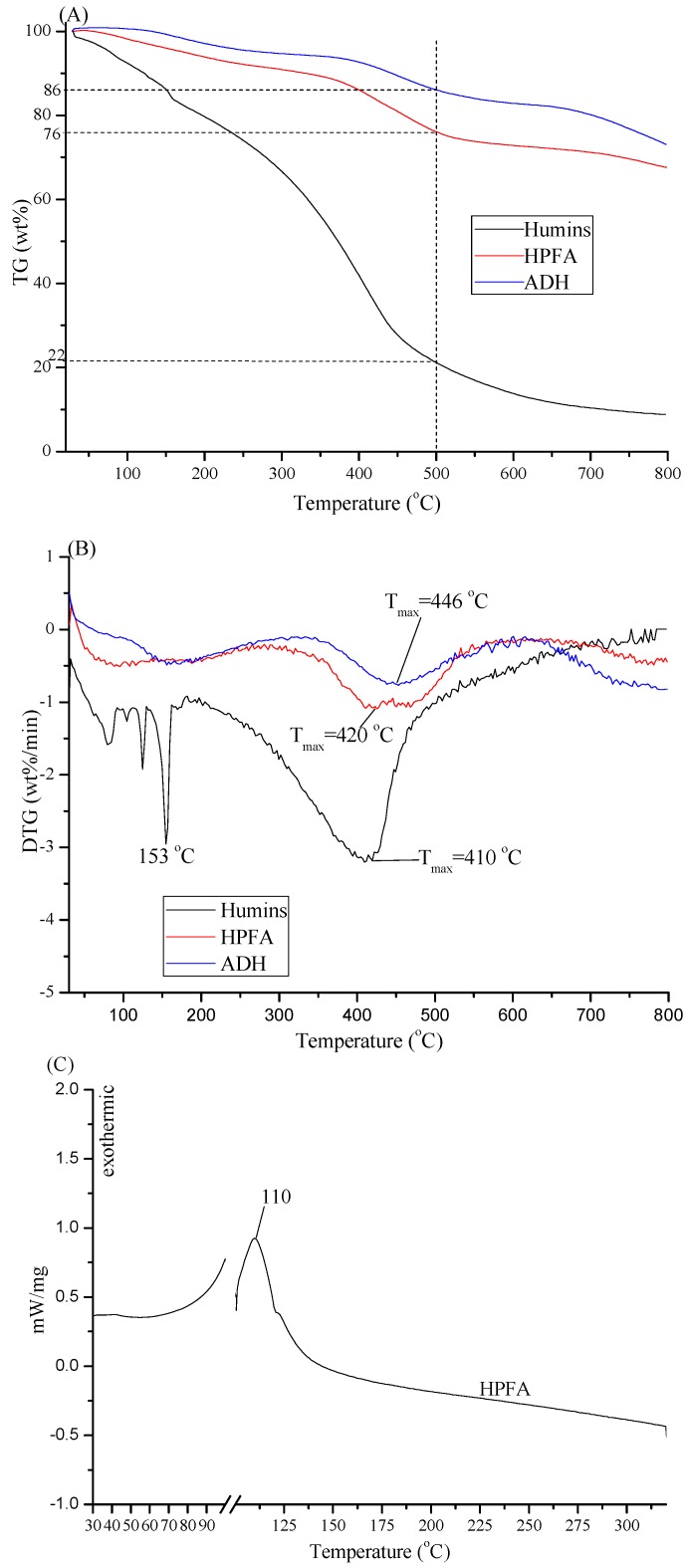
Thermal analysis spectra. (**A**) TG of humins, cured ADH and HPFA; (**B**) DTG of humins, cured ADH and HPFA; (**C**) DSC of HPFA.

**Figure 3:**
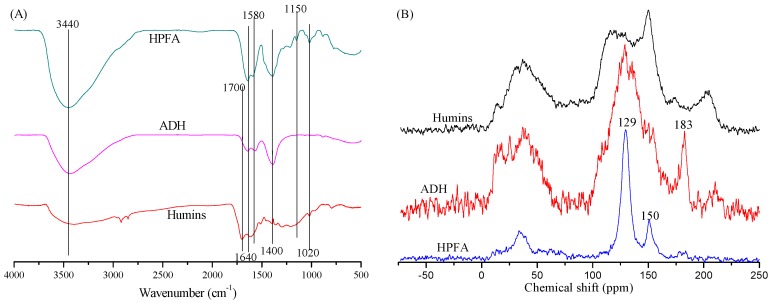
(**A**) FT-IR spectra of humins, cured ADH and HPFA; (**B**) C^13^ NMR spectra of humins, cured ADH and cured HPFA.

Interestingly, the structure and properties of the humins changed significantly after the hydrothermal alkaline treatment, which was reflected by the SEM (Figure 1), thermogravimetric (Figure 2), FT-IR and solid C^13^ NMR (Figure 3) analysis. Figure 1 illustrates that the humins are solid particles comprised of microspheres with diameter = 5–12 μm, whereas the morphology of the cured ADH is a bulk solid with wrinkle surface. The thermogravimetric analysis results shown in Figure 2 indicate that the cured ADH is thermally more stable than humins. When the TG heating temperature reaches 500 °C, approximately 86 wt % of the cured ADH remains, which is much higher than that of humins, approximately 22 wt %. This is also reflected by the DTG analysis, since a higher maximum derivative peak temperature (*T*_max_) but a related lower derivative value was observed for ADH as compared with humins. The maximum derivative peak temperature (*T*_max_) of the humins, ~410 °C, was lower than that of cured ADH, ~446 °C. However, the absolute derivative value at *T*_max_ of humins > |−3| wt %/min, is much more than that of cured ADH, <|−1| wt %/min. The improved thermal stability of cured ADH results from the structure change of humins after the hydrothermal treatment, i.e., broken of C–O (alcoholic hydroxyl, ether and acetal aliphatic) linkages and formation of aromatic structure as discussed below. Generally, the bond dissociation energy of C–O is much lower than that of the formation energies of aromatic C–H and C=C [17], and the C–O structures are thermally less stable than the aromatic structures [18,19].

The FT-IR and C^13^ NMR analysis shown in Figure 3 illustrated the change in functional groups. Humins contain abundant C–O bonds, and it has been reported that the humins consist of a network of 60% furan rings linked with 20% ether or acetal aliphatic linkers [6]. Similar results were also reported by Orella, indicating that the C–O bonds of humins were comprised of ether, acetal, and alcohol groups, which can be seen in the peaks with wavenumbers between 1000–1200 cm^−1^ in the FT-IR spectrum [20]. In addition, it has been reported that the C–O bonds of humins were reflected by the peaks at around 1020 cm^−1^ and/or around 1160 cm^−1^ in FT-IR spectra [21,22,23]. Thus, the FT-IR peaks at around 1020 and 1150 cm^−1^ in Figure 3A demonstrated the existence of C–O bonds. Both peaks, however, significantly decreased in the spectrum of cured ADH, indicating the breaking of C–O bonds during the hydrothermal treatment. Ketones and/or aldehydes (reflected by the broad peak at around 210 ppm in C^13^ NMR spectrum) were important functional groups of humins, but the concentration of these groups decreased significantly in the cured ADH (seen Figure 3B). This was a result of the strong alkaline conditions, which can lead to a series of reactions with ketones or aldehydes, such as Aldol condensation, Cannizzaro reaction and Darzens reaction [24]. In addition, hydrothermal treatment of carbohydrates causes the formation of products containing carboxyl groups [19,25], which was also observed in this study indicated by a strong signal around 183 ppm of cured ADH in C^13^ NMR (Figure 3B).

**Figure 4:**
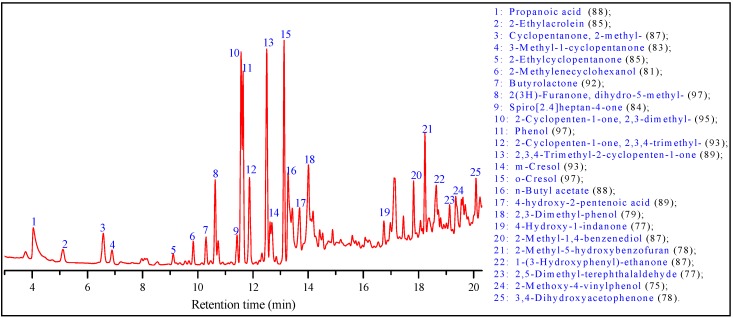
GCMS analysis of the methylene chloride extracted products in ADH solution. Values in parentheses indicate similarities (%) identified the NIST08 mass spectral data library.

Phenolic groups also existed in the cured ADH, which was illustrated by the peaks of 1640 and 1580 cm^−1^ (aromatic groups) and 1400 cm^−1^ (phenolic hydroxyl groups) in FT-IR spectrum [26,27], and the peaks at around 129 ppm (C of benzene ring) and 150 ppm (C of benzene ring connected phenolic groups) in C^13^ NMR spectrum. The formation of phenolics was also demonstrated by GC/MS analysis of the low molecular weight compounds in the ADH solution as shown in Figure 4. Organic acid, phenolics, ketones, esters, and other cyclic compounds were identified, in which phenolics are the major components. These identified phenolics include phenol, *m*-cresol, *o*-cresol, 2,3-dimethyl-phenol, 4-hydroxy-1-indanone, 2-methyl-1,4-benzenediol, 2-methyl-5-hydroxybenzofuran, 1-(3-hydroxyphenyl)-ethanone, 2-methoxy-4-vinylphenol, 3,4-dihydroxyacetophenone.

Apparently, alkaline-catalyzed hydrothermal treatment is an effective method for dissolving humins, and the ADH formed has improved thermal stability. The high phenolic content and homogeneous properties of the ADH solution make it a good candidate reagent for PF synthesis. The ADH solution, therefore, can be used as a petroleum phenol substitute for HPFA production. It is worth noting that the ADH solution cannot be directly used as adhesive owing to its low bonding strength (0.29 MPa), low dynamic viscosity (12.5 mPa·s), and low solid content (31.2 wt %) as shown in Table 1.

### 3.2. Polycondensation Reaction

In a polycondensation reaction, an equivalent amount of phenol and ADH reacted with excessive formaldehyde, which means ADH replaced about 50% of phenol to synthetize HPFA. The concentration of free phenol and free formaldehyde after the reaction was 0.2 wt % and 0.2 wt %, respectively (as listed in Table 1), indicating that polycondensation reactions with phenol, ADH and formaldehyde as reactants were successfully conducted. Compared with the ADH solution, the HPFA formed had an increased solid content, 45 wt %, and a decreased pH value, 9.4. The decrease of pH value is caused by the utilization of phenol (weak acid) and unavoidable adsorption of CO_2_ during the reaction. The dynamic viscosity of HPFA reached 112 mPa·s, which results from the increase of solid content and molecular weight in the polycondensation reaction. The cured HPFA has a smoother and more compact structure compared to the cured ADH as shown in Figure 1B,C, indicating that the polycondensation reaction improves the crosslink density among the constituents of HPFA [28]. This was also shown by the C^13^ NMR analysis shown in Figure 3B, since the peaks at 129 and 150 ppm of cured HPFA were sharper than that of cured ADH [26]. An improved crosslink density usually results in the increase of bonding strength [29]. Actually, the bonding of HPFA (0.83 MPa) was much stronger than that of ADH solution (0.29 MPa), but somewhat lower than that of certain traditional PF adhesive for wood adhesion (e.g., 1.1–1.3 MPa) [30,31]. However, the bonding strength of HPFA was higher than the bonding strength requirement (0.7 MPa) of Chinese National Standard (GB/T 14732-2006). Importantly, the HPFA was prepared by replacing phenol with low-value biorefinery byproduct humins, which has significant economic and environmental benefits.

### 3.3. Characterization of HPFA

Dynamic viscosity, bonding strength, pH value, free formaldehyde level, free phenol level, and solid content are six key indexes for wood adhesive. Table 1 also compares these properties of HPFA with Chinese National Standard (GB/T 14732-2006) requirements. All these indexes of the HPFA synthetized met the Chinese National Standard (GB/T 14732-2006) requirements for phenolic wood adhesive. Therefore, the synthesis of HPFA from humins is a potential pathway for converting low-value-added humins to useful wood adhesives.

Since the ADH is the main reactant for the synthesizing HPFA, the main functional groups of ADH and HPFA are very similar, and most of the bonds found in the ADH also appear in the HPFA (Figure 3A). For instance, the HPFA also contains phenolic groups according to the peaks at 129 and 150 ppm in C^13^ NMR spectrum (shown in Figure 3B), and the peaks around 1400, 1640 and 1580 cm^−1^ in FT-IR spectrum (shown in Figure 3A). The HPFA, however, has a strong intensity of phenolic groups but weak intensity of other groups (e.g., the carboxyl groups at around 180 ppm) comparing with ADH as reflected the C^13^ NMR spectra (shown in Figure 3B), as half of phenol was added during the HPFA synthesis, which decreased the intensity of other functional groups. In addition, the HPFA also has peaks at 1150 and 1020 cm^−1^ (C–O bonds) as humins in the FT-IR spectrum (shown in Figure 3A). These peaks were not observed in ADH and the other reactants, i.e., phenolics with formaldehyde. The C–O bonds, therefore, should be formed during the polycondensation reactions.

Curing temperature is an important factor in adhesive selection. In general, a relative mild curing temperature is advocated to lower the requirements of curing process. Based on the DSC analysis (shown in Figure 2C), the curing temperature of HPFA is 110 °C, which is much lower than that of PF adhesive (136 °C), lignin modified PF adhesive (157–188 °C) [29] and hydroxymethyl phenol-modified soy-based adhesive (140 °C) [27].

As discussed above, the thermal stability can be reflected by the remaining TG weight at the given temperature. When heating to 500 °C, 76% of cured HPFA remains, which is a slightly lower than that of cured ADH but much higher than that of humins (see Figure 2A). The thermal stability of the cured HPFA, therefore, is slightly lower than that of cured ADH, which is probably a result of the formation of unstable bonds (e.g., C–O groups). The cured HPFA, however, has better thermal stability comparing to humins.

**Figure 5:**
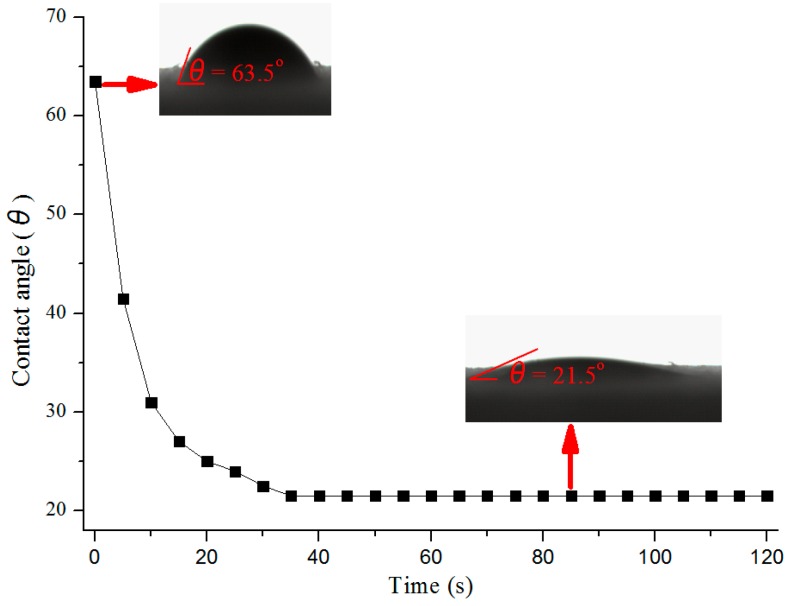
The contact angle of HPFA on pine wood.

Generally, the interaction between PF adhesives and wood involves mechanical interlocking and chemical bonding (e.g., van der Waals forces, dipolar interaction and hydrogen forces) [32]. As both the HPFA and the wood are rich in polar groups (e.g., hydroxyl group), dipolar interaction and hydrogen bonds are formed. The hydroxyl group in HPFA can be reflected by the huge peak at 3440 cm^−1^ in FT-IR spectrum (Figure 3A), while the hydroxyl group of wood can be found in cellulose and hemicellulose constitutes. The mechanical and chemical bond strength require the wetting and flow of the adhesive across and into the wood surface, and poor wetting reduces contact area and thus bond strength between adhesive and wood [32]. The contact angle is therefore very important, which determines the wetting of the wood surface, and a lower contact angle usually means greater wettability. Pine wood has a surface with good water wettability, since a control experiment showed that it absorbs water droplets within 20 s. Pine wood was selected as the substrate for the measurement of HPFA’s contact angle, as shown in Figure 5. The initial and equilibrium contact angles of HPFA on pine wood were 63.5° and 21.5°, respectively (see Figure 5). This equilibrium contact angle is relative low as compared with the equilibrium contact angles (e.g., 30°–70°) of commercial PF adhesive used for wood adhesion [33,34,35]. This low equilibrium contact angle indicates a desirable wettability of HPFA for the adhesion of pine wood.

## 4. Conclusions

Humins, low-value-added biomass hydrolysis byproducts, were discovered to be a good feedstock for humin-phenol-formaldehyde adhesive (HPFA) synthesis. A two-step process was developed for synthesizing HPFA: (1) degradation of humins to ADH through hydrothermal treatment, and (2) polycondensation reactions by substituting 50% of phenol with ADH. HPFA is a potential wood adhesive, with all the six key indexes (dynamic viscosity, bonding strength, pH value, free formaldehyde level, free phenol level, and solid content) meeting the Chinese National Standard (GB/T 14732-2006) requirements. In addition, the HPFA had a relative mild curing temperature and desirable wettability for pine wood.

## Figures and Tables

**Table 1:** Properties of HPFA and ADH solution.

Properties	ADH Solution	HPFA	Chinese National Standard (GB/T 14732-2006)
pH	13.2	9.4	≥7
Viscosity (mPa·s)	12.5	112	≥60
Solid content (wt %)	31.2	45	≥35.0
Bonding strength (MPa)	0.29	0.83	≥0.7
Free formaldehyde level (wt %)	-	0.2	≤0.3
Free phenol level (wt %)	-	0.2	≤6
